# Diagnosis of diabetes mellitus and living with a chronic condition: participatory study

**DOI:** 10.1186/s12889-018-5637-9

**Published:** 2018-06-05

**Authors:** José Adailton da Silva, Elizabethe Cristina Fagundes de Souza, Ana Gretel Echazú Böschemeier, Camyla Cristina Maia da Costa, Héllydade Souza Bezerra, Eva Emanuela Lopes Cavalcante Feitosa

**Affiliations:** Federal University of Rio Grande do Norte, Natal, Brazil

**Keywords:** Chronic illness, Participatory research, Health promotion, Personal autonomy

## Abstract

**Background:**

Diabetes mellitus is one of the most serious chronic *illnesses* in the world *due to* its prevalence, economic and social effects, and negative impact on the quality of life of the affected people. The diagnosis implies changes in life habits especially related to feeding, physical activity, and constant self-care, requiring greater personal autonomy.

**Methods:**

This study aims to understand how individuals living with diabetes deal with the recognition of the chronic condition in their health care practices. This is a participatory research with a qualitative approach focusing on reflexivity. Sixteen *people* with diabetes mellitus were intentionally chosen, and qualified to participate in the study. The selected methodology allowed the constitution of life stories and focused *on* the multiple ways human beings deal with their illnesses.

**Results:**

The participants attended eight closed group meetings, with an specific methodology which benefited them to retrieve their own history as well as the multiple experiences to deal with the disease, here called Strategic Health Promotion Group (SHPG). The data produced and the dialogue between researcher and researched subjects were related to three major thematic perspectives: I) recognizing diabetes II) living with diabetes III) exercising personal autonomy. This work contains the meanings attributed to the Perspective I from which the following three categories emerged: The impact of the diagnosis, the denial of the illness, and the acceptance of the illness. It was observed that the diagnosis of a chronic illness generates a multiplicity of feelings, moving through narratives of complications and death events shared between generations. The participants expressed feelings related to denial or acceptance of the chronic condition which required an active adaptation exercising. From the current diagnosis, it was observed that new signs were added to the person’s existence, influencing their habits, health care practices and quality of life.

**Conclusions:**

The emotional aspects of subjects diagnosed with diabetes mellitus strongly influence the acceptance or denial of the illness, interfering in their personal adherence to treatment. As a chronic condition, involving life-longing care practices, which intervenes in therapeutic participation, it is indispensable to respect and to encourage the personal autonomy of the subjects.

## Background

Chronic non-transmissible diseases represent a major problem in the world, being the main cause of death today [1]. Diabetes mellitu*s* is one of the most worrying chronic diseases for its major economic and social impact, reported as responsible for 11.6% of the health care expenses worldwide in 2010 [2]. According to the World Health Organization [3], in 2014, a total of 422 million adults had diabetes and in 2012 there were 1.5 million deaths caused by this illness.

Diabetes *mellitus* is defined as a syndrome caused by several etiologies and is characterized by a metabolic dysfunction with a degenerative potential that involves energetic sources resulting from changes in the production, secretion and/or inability of the insulin to adequately exercise its effects. It is a chronic condition that requires the subjects living with the illness to have a continuous self-management of the lifestyle and adaptation to the illness [4].

Diabetes mellitus is often considered a silent illness and linked to poor health care. In fact, 46.5% of the affected people are unaware of their condition [5]. Thus, the news of having diabetes is often abrupt and may be accompanied by feelings of denial and/or difficulty in the treatment participation, which involves important changes in lifestyle.

The projections for diabetes mellitus are worrying. Currently, 10% of the world population lives with the illness and it is estimated that, by 2025, 300 million people will be affected. At that time, 75% of people with diabetes will be residents of developing countries, as a 170% increase in new cases is estimated for these countries, while an increase of 42% is expected for in developed countries [6].

The statistics in Brazil are close to the world average. However, it is estimated that by 2030, the country will occupy the 6th position in the world ranking, with 11.3% of the population affected by the illness [2].

However, more than preventive actions, the health care system needs health promoting actions that impact on the quality of life of the subjects already affected by the illness. It is necessary to stimulate a greater autonomy for self-care actions and participation into the required treatment, strategies that go beyond the use of medications.

The main interferences into different diabetes types of treatment are caused by the negative emotional reactions that arise before the need for permanent care to control the illness. The subject’s emotional background can set difficulties to the adoption of self-care actions and participation in the treatment [7].

The treatment of diabetes encompasses a number of factors, some of which are specific, other global. Overall, they all involve a permanent education and the modification of a lifestyle from the very moment that the disease has been diagnosed. For subjects, this includes submitting to rules that are not always well accepted, such as healthy eating, correct and regular use of medication, including oral anti-diabetic drugs and/or insulin, and self-monitoring of blood glucose. Living with a chronic condition can be very threatening because it affects all the signs of the subjects, changing their routine and their relatives’ routine [8].

The results presented in this article are a part of the research Project “Health Promotion Strategies: Autonomy challenges in subjects living with Diabetes Mellitus”, from the Collective Health Department at the Federal University of Rio Grande do Norte. Its main objective is to “compose health promotion strategies in a direction to stimulate the autonomy and health care of people living with diabetes”. For doing so, it was initiated the creation of a Strategic Health Promotion Group (SHPG). In this article, results are presented around the objective of “sharing the experiences of people living with diabetes as well as how they build and fortify their autonomy, facing the necessity of lifestyle changes and their participation in the therapeutic and health care decisions”. The importance of this study for public and collective health is linked to the need of recognition not only of the subject’s own perceptions and life strategies, but also to the significance of searching new forms of understanding their condition, through dialogue, as well as the discomfort related to the impact of the diagnosis and its daily treatment. This project also intends to elaborate strategies directed to therapeutic participation, proposing innovative ways of dealing and taking care of the health of subjects with a chronic condition.

The working groups of people living with diabetes are generally associated to linked diseases like high blood pressure and are very common at Basic Health Care. However, they are frequently directed only to a biomedical approach consisting of: prevention, control and treatment of diseases [9], diverging from the emancipatory actions of subjects that are proposed by Health Care Promotion actions. In the Brazilian Health System, groups like the SHPG - that is described here - could help to qualify the processes of Basic Health Care, consolidating its attributes and fortifying Health Care Promotion actions in accordance with the principles and guidelines of the National Health Promotion Policy.

The knowledge about the perception of patients who live with diabetes is important because of the psychosocial discomfort and the continuous treatment hinder the participation of these subjects to their new way of life. The present study aims to understand how subjects with diabetes deal with the fact that they are chronically ill and how being aware of their situation influences the way they take care of their health.

## Methods

This study is a participatory research with a qualitative and reflexivity-centered approach. It followed the recommendations of the Research Ethics Committee (REC) from the Onofre Lopes University Hospital (HUOL) at the Federal University of Rio Grande do Norte (UFRN).

A participatory research strategy was adopted considering the production of knowledge guided by shared care, where the subject who needs health attention, in this case, people living with a diabetes mellitus condition, are at the center of the stage. An epistemological, clinical and methodological approach based on shared care stimulates the preeminence and autonomy of the subjects, considering the individual, familiar, social and cultural dimensions of the person who is under its view and treatment [10]. Therefore, a participatory research approach shows its dynamism and capacity of being a strategy for social change, relying on the mutual collaboration among the subjects who take part on the process [11].

Following this perspective, we adopted the participatory research strategy described by Passos et al. [12]: in this proposition, knowledge and awareness are first developed in each group,for subsequent collective comprehension, being the responsibility of the researchers to take care of their own intervention by a reflexive approach, characterizing the ethical-political nature of the research.

Subjects with both type 1 and type 2 diabetes were intentionally chosen to participate in the group as long as they agreed with being part of the experience, as long as they were monitored by a health unit located in Santa Cruz, a small town from Rio Grande do Norte – Brazil, which was the research field qualified to participate in the study. The total number of voluntary participants was justified as an intentional sample derived from a specific population of 70 (seventy) people diagnosed with diabetes mellitus belonging to this health unit. The minimal number defined to compose the group was of 12 subjects, being the maximum a number of 25 subjects. To define the minimum and maximum values of the sample there was a specific operational criteria that would allow the participants to feel assisted, to communicate in an effective way, to acknowledge and recognize each other and, finally, to build the particular dynamics of the group identity [13]. The sample size should also allow the coordinators to feel comfortable during the process of assessing the communication of the group members. We also considered the potential loss of participants during the programmed 8 (eight) sessions, by spontaneous quitting or personal impediments. The eight encounters treated specific topics chosen by the individuals participating in the group. This closed group is in the category of the denominated SHPG – Strategic Health Promotion Group.

The encounters took place between May and August 2017, with a specific methodology oriented to capture the life stories and multiple experiences of the subjects. Those experiences were initially related to the illness, but the narratives moved beyond it to connect this experience with other aspects of their life’s complexity. Each meeting was defined based on a chosen topic coming from the previous one. The role of the researcher in the coordination of a participatory group is to mediate the dialogues between the subjects that are a part of the research, without missing the objective of contributing with the shared management of the participants.

This type of group management has been developed in researches about mental health in Brazil and Canada [12, 14]. Those experiences have taught us the importance of dissipating the centrality of the researcher – observer to evidence the collective realm where knowledge construction through shared experiences manifests itself. In such approaches, the shared management brings up the necessary opening for the production of each group’s identity, and, consequently, the production of a shared group autonomy.

In terms of the techniques used to collect the narratives, it is necessary to point out that the meetings were audio recorded and the speeches were transcribed literally and in their complete versions. The produced data has been classified into three main thematic axes composed by the following dialogue stages: I) recognizing diabetes; II) living with diabetes; and III) exercising personal autonomy. The current study contains the analysis of the perspective I “Recognizing diabetes”, which deals with the impact of the diagnosis and the process of acceptance and denial of the chronic condition. For the sake of a better organization, we chose to have the statements that compose the perspective I in categories defined upon the objective of the study and quoted above.

The content of the data was analyzed under Minayo’s perspective [15], and under the directions of a reflexive approach, which in this study is considered as the ability of the subjects to monitor their own actions and their own desires through traditional principles of promoting the dialogue. For researchers, the reflexive dialogue can be established within themselves and their subjective preferences as well as with the collective experience, pursuing an attitude of continuous anxiety about one’s own actions, the preconceptions involved in each decision, and the methods used to filter, to control, to define, and to guide the social processes [16].

Written informed consent was obtained from all participants. In order to maintain confidentiality, each participant was identified by the name of an Ancient Greek city. Despite its geographical distance, this country has a particular relevance in the etiology of two central words that guide this research experience: diabetes (διαβαίνειν) and autonomy (αὐτόνομος). In this study, the participants’ names are: Corfu, Fira, Mykonos, Meteora, Veria, Atenas, Heraklion, Creta, Rhodes, Castória, Micenas, Patras, Delfos, Zakynthos, Epidauros and Volos.

## Results

All 16 (sixteen) participants live with Type 2 Diabetes Mellitus, of whom 12 (twelve) are females and 4 (four) are males aged from 57 to 90 years.

Based on the perspective I “Recognizing diabetes” and on the objectives of this study, the results were organized into three categories discussed below, namely: the impact of the diagnosis; the denial of the illness and the acceptance of the illness.

### The impact of the diagnosis

This category shows how subjects reacted to the discovery of their chronic condition, which was mostly unexpected. The impact of the news caused several reactions, a mix of emotions and feelings, such as despair, preoccupation, unrest and even panic, when the information was disclosed by means of a diagnostic test or a health professional:

*“I discovered it at age 42... I was a bit overweight. The doctor said: you are diabetic! What a shock! I almost died!”* (Fira).

*“I panicked. I thought I was never going to be normal anymore in my life. I panicked! Do you know what panic is?”* (Zankythos).

As diabetes sometimes has a silent character, in some cases it can be discovered with important previously installed complications, and this can lead to the feeling of anger and revolt at the moment of diagnosis, as shown in the speech of Patras.

*“At first I felt anger, I was angry at the time, because when I discovered I was already harmed, I already had my vision affected, then at the time I felt revolted, angry”.* (Patras).

For Heraklion, however, the reaction to the chronic condition was not immediate, perhaps due to the non-correlation of the diagnosis of diabetes with a chronic condition:

*“At that time (the diagnosis) I confess that I did not feel anything... only with time I started to feel the difficulties and started to get more... I wouldn’t say unhappy, but worried!”* (Heraklion).

Still on the impact of the diagnosis, we also detected a change of attitude of some participants in relation to the recognition of diabetes. Some expressed certain predictability in relation to the illness, especially because of heredity from the histories of complications and deaths shared between generations, as we can see below:

*“I did not suspect (of the illness) but I had doubts, because of the family, my father died at 45, most of Dad’s family all died of diabetes... everything is hereditary”.* (Mykonos).

Other speeches also drew attention to the recognition of hereditary factors in the diagnosis of the illness. However, there is again concern and fear about the prognosis, when they compared their situation to that of people they had heard of in their life experiences:

*“I come from a diabetic family, my brothers and sisters have it. One of them is already dead”.* (Meteora).

*“I’m also from a diabetic family. Most of my uncles had problems (with diabetes) and died”.* (Véria).

*“My brother died of diabetes, he lost both legs, so I’m afraid because I remember this when my diabetes (glucose) gets too high...”* (Volos).

In this category, it was observed that the dialogues in a group caused important reflections in the way each participant faced the diagnosis, which can influence the different stages of acceptance and denial of the chronic condition and in its ways of carrying out self-care as well as in dealing with institutional health care.

### The denial of the illness

We imagine that the way to deal with the diagnosis and the new ways of carrying on with life directly influences the acceptance or denial of the illness. This category is composed by the dialogues concerning the non-acceptance of the chronic condition attributed, mainly, to the limitations of daily life implied by having to live with a long-term illness.

Denial is present in the reactions of dissatisfaction exposed by Castória.

*“You feel unhappy by having diabetes. There are days that I meditate a lot, there are times when I am alone, sometimes I cry, then I try to visit someone; I want to get that thought out of my head. I cry because of diabetes and because of the difficulties too”.* (Castória).

For Veria, the dissatisfaction, revolt and non-acceptance comes from the lack of the indicators control of the illness caused by many factors:

*“I was diagnosed four years ago ... but I cannot control it, I could never control it, I take insulin, but I cannot control it ... I thought it was a simple thing. I thought I was going to control it”.* (Veria).

Other participants, in the dialogue in a large group, end up expressing the dissatisfaction with the illness resulting from abrupt changes in lifestyle, not always tolerated, especially when related to food:

*“It’s very annoying to live with diabetes, you cannot eat everything, and you have to be on a diet”.* (Heraklion).

*“I got like this because it is an illness that forbids us to eat everything, I miss it, especially the sweet stuff”.* (Epidauros).

On the other hand, the participants also take the group to reflect on stigma towards the illness as well as on the fact that seeking to adapt to the illness, doesn’t always mean accepting it:

*“I feel sad because of this diabetes, I tell everyone it was a nickname they gave me, I do not say that I am diabetic, it was a nickname they gave me”.* (Micenas).

*“No one gets rid of this. Diabetes is forever. But, I control it and I live”.* (Volos).

Thus, living with diabetes is complex and non-acceptance can come from multiple factors, all of which permeate the meanings people give to their lifestyle habits.

### Acceptance of the illness

Acceptance of the illness, the last category analyzed, may be directly related to the control of the illness and strategies for overcoming and living with the new chronic condition. It is evident that there is an understanding of the importance of self-care for their well-being and on their health-illness process:

*“I take the medicine on time, I do not eat food I’m not supposed to eat, we cannot eat them! We need to adapt”.* (Corfu).

*“You have to try to do those physical activities that are good for diabetes: you have to try to walk, exercise, swim, it’s extremely good for us”.* (Rhodes).

Patras’s speech also contributes to the understanding that by controlling the illness, acceptance becomes more feasible, as an intrinsic relationship between accepting-controlling and controlling-accepting.

*“Diabetes is an illness that we will live with and we have to try to be happy... under control! We need to try to control every day more... food, physical activity, medicine, taking them correctly, always up to date with the medication”.* (Patras).

In this category, reflexivity is also raised about the exchange of experiences of the group. The dialogues with the participants could facilitate the acceptance of the chronic condition, as it’s seen in the speech of Epidauros:

*“Today I think I’m more relaxed, I think I’m more optimistic, to know what diabetes is, how we should live together and what to do to have better health”.* (Epidauros).

Accepting a chronic illness is a gradual process where the subjects first become aware of their situation, by this means, facilitating their adaptation. This contributes to a better quality of life and decreases the risk of complications related to the illness.

## Discussion

The discourses of the participants regarding the impact of the diagnosis made it possible to perceive that a mixture of feelings emerge. They include preoccupation, panic, and even anger and consequently denial of the condition. This was also observed in another study that identified the existence of a relationship between emotion and diabetes, where one directly influences the other [17]. It is also known that the emotional impact generated by the diagnosis and its entire burden triggers the complications of diabetes mellitus [17].

In this same perspective, some studies have pointed out a greater vulnerability of people living with chronic conditions to the emotional instabilities that can vary from mild to serious problems, such as severe depression, and that would be associated with the therapeutic rigour of the illness [18].

It is important to understand that diabetes has a negative impact on the quality of life of the subjects, especially due to the emotional changes, the limiting situation, and the process of (non-)acceptance of the illness. A study carried out in Brazil that measured the quality of life of patients, identified that among the subjects studied, physical and emotional factors were the most determinant for their quality of life, and the emotional impact occupied the second place, corresponding to a value of 77.2%. The authors explained that diabetes mellitus interferes in the patients’ quality of life in a negative way [19].

Still on the diagnosis of Diabetes Mellitus, some authors [17] mention that the diagnosis is made through routine tests or through the suspicion of symptoms of the illness. They also affirm that, as in the present research, the disclosure and confirmation of the diagnosis generates many feelings before the new chronic condition.

In the context of acceptance, denial, and the impact of diagnosis, we can describe the stages of mourning explained by Elisabeth Kubler-Ross [20] in her book “On Death and Dying”: denial, anger, bargaining, depression and acceptance. Some studies [21, 22] have shown that the impact of the diagnosis of a chronic illness resembles the stages of mourning, as it is always very difficult and affects the self-image and self-esteem. People that receive the diagnosis, just as mourning, go through several stages where feelings can go through several phases. The stages of mourning should be used in a flexible way and help a comprehensive understanding of the subjects diagnosed with a chronic illness [21].

Fear was a common reaction in the dialogues of the participants of this study, especially when they reported traumatic family experiences with complications and deaths caused by diabetes. Some studies pointed out that sometimes the awakening to the illness can cause bad feelings when, for example, the patients are aware of histories with traumatic prognoses such as death or amputations, and because of this, they tend to fear the repetition of those events with risk of tragic ends, such as those observed in relatives or close friends, especially arising from the fear of chronic complications of the illness [23, 24].

The acceptance of a chronic condition is a result of a transformation that takes place gradually in the behavior of the subjects, moving in the direction of a greater awareness and adaptation to the illness. These aspects directly contribute to their responsibility towards their overall health state. When patients accept their condition, they find an inner peace, thus favoring acceptance and better adaptation to their chronic condition [8].

However, the difficulty of accepting a chronic condition brings subjects to a particular dialectic dynamic in their lives. Accepting the chronic condition requires that the person recognizes and learns somehow to live with the discomfort and pain generated by the restrictions imposed by the new habits. It is necessary to reflect on alienated acceptance or adaptation to new habits, always re-evaluating the health practices so as not to stigmatize subjects, because words such as “patients, diabetic person, carrier” reduce the subjects to objects and as they are submitted to passivity. Accepting treatment, in a more minimalist conception, “implies recognizing oneself as having a significant limitation, determined by a chronic illness. It implies, therefore, a loss of autonomy” [25]. The sense of being stigmatized was exalted in this study. In this sense, autonomy is fundamental even to favor therapeutic participation into the new ways of dealing with life.

During the dialogues with the group, we also noticed how the impact of the diagnosis and new ways of living influence on the emotional state of those subjects. The fact of having to live with a chronic illness causes discomfort and dissatisfaction because the condition requires a complex and long-term treatment which impacts on the whole social network in which the subject is inserted. This demonstrates the importance of the exchange of experiences in the pursuit for greater autonomy.

It is known that exchanging experiences also influences on a better acceptance of chronic conditions, because when it is possible to live with the illness, there is time for a greater acquisition of knowledge, generating greater ease in the management of the treatment [18]. Thus the experience acquired individually is strengthened when transmitted to others through group dialogues that address individual experiences in search of collective pacts.

Therapeutic participation is a conditional factor for improving the quality of life of subjects with chronic diseases. Studies have indicated that the therapeutic success in patients with diabetes *mellitus* is linked to the extent to which the subjects act and commit themselves for their well-being, as for example by monitoring their glycemic status, changing their life habits and making correct use of medications. Counseling about the illness and its treatment is very important for therapeutic participation in the care of the subjects, improving their life quality [26–28].

The group experience can also potentialize changes in lifestyle. However, changes in life habits are complex; it is a difficult and slow process, particularly with regard to food. Eating habits are related to cultural, economic and social factors [25]. We identified in the speeches of the participants that food is one of the most difficult factors to control the illness. Other studies [19, 29] have stated that the negative impact on the quality of life is influenced by the new lifestyle, which should be adopted, and especially because people with diabetes need more food restriction, which has a greater impact on therapeutic participation and health care. Thus, food re-education is one of the greatest challenges faced by some people with diabetes mellitus, where negative feelings related to food control occur, such as the difficulty to meet the goals set by the health team and consequent frustration [18].

Recognition of diabetes, in its broadest sense, emerges not only through the knowledge of clinical diagnosis, but also through the repositioning of the subjects in their ways of living and taking care of health. It goes through reflexivity, looking and re-signifying the perception of themselves and their social network, identifying their beliefs, values and establishing a relationship of mutual support in the search for autonomy. It is known that recognizing oneself in the condition of living with a chronic illness is fundamental for the good performance of self-care actions and, consequently, for their participation on treatments [7]. It is also fundamental to guarantee the control of their own lives, so that the person may be able to deal with the limitations that are imposed by the diabetes mellitus, with co-responsibility as a fundamental aspect for the success of the treatment and for the quality of life [30]. Participation through the exchange of experiences in the SHPM was able to influence this context in a positive way.

Providing autonomy to the subjects means to consider them as owners of conceptions and experiences that directly influence their relationship with both health professionals and their own health-illness process. Thus, the experience of illness and self-care is taken into account [21, 31].

Furthermore, it is important to work and encourage the personal autonomy of subjects who live with diabetes because they are full of experiences that influence their ways of living with the chronic condition. Participatory studies are still insufficiently explored in the context of groups of people living with diabetes from the perspective of autonomy. Moreover, further studies are needed to deepen the theme and to make it possible the construction of new propositions. It is also necessary to apply the SHPG in multi-centric studies to improve the analysis related to the subject’s mutual sharing of illness experiences and narratives in therapeutic participation and health care, especially on the stages of acceptance and denial of the illness.

## Conclusions

This study allowed us to observe that the emotional aspects involved in the diagnosis of diabetes *mellitus* can influence whether or not the illness is accepted. The way in which the subjects recognize diabetes and re-evaluate their habits influence their participation into treatment, because this is a chronic condition in which changes are often marked by their duration and by the risk they offer.

The denial of the chronic condition results in a greater difficulty in developing self-care, influencing the lack of participation into the different approaches of treatment.

Denial of the illness seems to be more related to the difficulties in controlling the illness resulting from changes in lifestyle. It is a difficult process for the subjects and requires a slow adaptation because an appropriate treatment needs the understanding of the implications of such changes in the subjects’ lives.

With regard to the acceptance of the chronic condition, we observed that it does not correspond to a static phase, but to a process of transformation that happens gradually. The subjects need to have a greater understanding about their personal recognition and their ways of dealing with health.

Giving voice to the participants as holders of the experiences affect the research process at its core. Participatory research in the light of reflexivity transformed the reality of both participants and researchers, overcoming the dichotomy between subjects and objects investigated. The possibility of a broader participation allowed the subject’s collective repositioning, transcending the automatic collection of data into strategies of knowledge production and practices related to self-care and shared care, based on the exchange of experiences.

In conclusion, we can affirm that the process of accepting-controlling the illness favors a better adherence to treatment, strengthening the personal autonomy inasmuch as life quality. Therefore, it is indispensable to respect and encourage the personal autonomy of the subjects, making them co-responsible for their treatment.

## Abbreviations

SHPG: Strategic Health Promotion Group (SHPG)
